# A phylogeny of the genus *Limia* (Teleostei: Poeciliidae) suggests a single-lake radiation nested in a Caribbean-wide allopatric speciation scenario

**DOI:** 10.1186/s13104-021-05843-x

**Published:** 2021-11-25

**Authors:** Montrai Spikes, Rodet Rodríguez-Silva, Kerri-Ann Bennett, Stefan Bräger, James Josaphat, Patricia Torres-Pineda, Anja Ernst, Katja Havenstein, Ingo Schlupp, Ralph Tiedemann

**Affiliations:** 1 Unit of Evolutionary Biology/Systematic Zoology, Institute of Biochemistry and Biology, University of Potsdam, Karl-Liebknecht-Straße 24-25, Haus 26, 14476 Potsdam, Germany; 2 Department of Biology, University of Oklahoma, 730 Van Vleet Oval, Norman, OK 73019 USA; 3 Department of Life Sciences, The University of the West Indies (Mona Campus), Kingston, Jamaica; 4 German Oceanographic Museum (DMM), Katharinenberg 14-20, 18439 Stralsund, Germany; 5 Caribaea Intitiative and Université Des Antilles, Guadeloupe, Kingston, Jamaica; 6 Museo Nacional de Historia Natural Prof. “Eugenio de Jesús Marcano”, Avenida Cesar Nicolás Penson, 10204 Santo Domingo, República Dominicana

**Keywords:** Cytochrome *b*, Island biogeography, Fresh water fish, Phylogeny

## Abstract

**Objective:**

The Caribbean is an important global biodiversity hotspot. Adaptive radiations there lead to many speciation events within a limited period and hence are particularly prominent biodiversity generators. A prime example are freshwater fish of the genus *Limia*, endemic to the Greater Antilles. Within Hispaniola, nine species have been described from a single isolated site, Lake Miragoâne, pointing towards extraordinary sympatric speciation. This study examines the evolutionary history of the *Limia* species in Lake Miragoâne, relative to their congeners throughout the Caribbean.

**Results:**

For 12 *Limia* species, we obtained almost complete sequences of the mitochondrial cytochrome *b* gene, a well-established marker for lower-level taxonomic relationships. We included sequences of six further *Limia* species from GenBank (total N  = 18 species). Our phylogenies are in concordance with other published phylogenies of *Limia*. There is strong support that the species found in Lake Miragoâne in Haiti are monophyletic, confirming a recent local radiation. Within Lake Miragoâne, speciation is likely extremely recent, leading to incomplete lineage sorting in the mtDNA. Future studies using multiple unlinked genetic markers are needed to disentangle the relationships within the Lake Miragoâne clade.

**Supplementary Information:**

The online version contains supplementary material available at 10.1186/s13104-021-05843-x.

## Introduction

The Caribbean is considered one of the most important global biodiversity hotspots [1]. The largest biodiversity is found in the Greater Antilles (Cuba, Hispaniola, Jamaica and Puerto Rico) where a remarkable diversification is observed in freshwater fishes [2–5], amphibians [6, 7], reptiles [8, 9], invertebrates [10–12] and plants [13, 14], putatively driven by a complex geological history, environmental heterogeneity, and the tropical climate [15, 16].

Adaptive radiations typically occur when a set of open niches becomes available because of a key innovation or the arrival of a founder species, which subsequently differentiates to occupy these niches [17]. Many classical examples are linked to islands, as Darwin’s Finches on the Galapagos islands, all of which go back to a single ancestor [18–20]. Research on Darwin’s Finches also highlighted the role of hybridization in speciation [21]. Other well-explored radiations include Hawaiian silverswords [22–24] and Hawaiian honeycreepers [25]. In all these examples, molecular evidence has played an important role in understanding the evolutionary processes of speciation. Probably the best-known examples from the Caribbean region are *Anolis* lizards [26] and *Eleutherodactylus* frogs [27].

Poeciliidae are freshwater livebearing fishes that have experienced an enormous radiation in aquatic environments of the West Indies with three endemic genera (*Girardinus*, *Quintana* and *Limia*) distributed in the Antilles [3, 4, 28, 29]. The Caribbean is also the site of two lesser known radiations in isolated inland lakes, both of which involve fishes of the genus *Cyprinodon* [30–33]. These Caribbean fishes share many characteristics with the most prominent example of radiation in freshwater fishes, the cichlids in lakes of the Rift Valley of East Africa, where each lake has produced a distinct cichlid fauna [34–36]. One of the important drivers for speciation in these fishes seems to be feeding specializations [33, 37, 38]. Furthermore, as generally predicted from the theory of island biogeography [39] and recently empirically confirmed for island birds [40, 41], the number and diversity of species in both the Rift Valley lakes and Greater Antilles correlates with the size of the habitat.

Among livebearing fishes of the Greater Antilles, the origin of the different lineages and species composition within each genus may show peculiar patterns [39, 42]. *Limia* is part of the unique freshwater fish fauna of the Greater Antilles. It is found in most freshwater habitats in Hispaniola, ranging from hypersaline lagoons to relatively cool mountain streams [43, 44]. *Limia* species are generally feeding generalists [2, 45, 46]. Their distribution indicates a radiation on Hispaniola [2, 47], with 19 of the 23 known species found on this island [46, 48] (Additional file 1). By contrast, on Cuba, Jamaica, and Grand Cayman, only one species each is found [28, 44, 49]. Within Hispaniola, nine *Limia* species (*L. fuscomaculata*, *L. garnieri*, *L. grossidens*, *L. immaculata*, *L. islai*, *L. mandibularis*, *L. miragoanensis*, *L. nigrofasciata*, *L. ornata*) have been described from a single site, Lake Miragoâne. This lake is one of the largest freshwater lakes in the Caribbean and is located in the southwestern part of Haiti. It comprises an isolated, endorheic drainage [50]. A *Limia* radiation there was hypothesized by Rivas [2] and has received renewed attention through the description of two new species from the lake [43, 45]. However, few studies have examined the evolutionary history of the fishes found in Lake Miragoâne.

Without specific attention to Lake Miragoâne, some studies of *Limia* have resolved the general phylogeny of the genus*.* Current literature suggests *Limia* to form a monophyletic group with the genera *Pamphorichthys*, *Mollienesia*, *Micropoecilia*, and *Poecilia*, with *Limia* as sister taxon to *Poecilia* [51–53]*. Limia melanogaster* is the most basal species, branching off early and colonizing Jamaica [2]. *Limia melanogaster*’s divergence was followed by the colonization of Hispaniola, where the species diverged into over 20 recognized species [44]*.* Nested within the species native to Hispaniola are *L. vittata* and *L. caymanensis* [2, 54] which are the only species native to their respective islands, Cuba and Grand Cayman [28, 44]. Most previous analyses target only a few species [52, 53, 55]*.* The most comprehensive phylogeny to date used nine species of *Limia*. Among them were only two native to Lake Miragoâne, *Limia nigrofasciata* and *Limia islai* [2, 44, 46], such that Riva’s hypothesis of a local radiation within Lake Miragoâne [1] could so far not been tested.

Our study comprises 18 out of 23 currently recognized species of *Limia*. It is particularly novel regarding its more comprehensive sampling of Lake Miragoâne, including five of its native species*.* We expected that if a local radiation event did occur in Lake Miragoâne, those species native to the lake should form a monophyletic clade.

## Main text

### Materials and methods

#### Sampling

Ingroup sampling consisted of 67 individuals representing 18 species of *Limia* (Additional files 2, 3). Twelve *Limia* species were obtained from wild-caught populations. Sequences from six *Limia* species were obtained from GenBank: *L. garnieri*, *L. melanonotata*, *L. pauciradiata*, *L. rivasi*, *L. versicolor*, and *L. sulphurophila*. Outgroup sampling consisted of eight individuals representing three species of *Poecilia*, the sister taxon to *Limia* [28, 44, 55]: *P. dominicensis* [45], *P. hispaniolana* [56], both endemic to Hispaniola, and *P. mexicana* from the Atlantic side of Mexico. We used four to five individuals per species, except in cases where sampling was limited.

#### Molecular methods

We targeted the mitochondrial (mt) cytochrome *b* gene, a well-established marker for lower-level taxonomic relationships as in recent radiations (see [57] for a fish example) for which we obtained an almost complete sequence.

Genomic DNA was extracted from muscle tissue using a cetyl trimethylammonium bromide (CTAB) protocol [58]. DNA concentration was measured using a NanoDrop ND-1000 and ranged from 2.7 to 120 ng/µl. Via Polymerase Chain Reaction (PCR), we amplified 1127 bp of the mitochondrial cytochrome *b* gene*.* Primers and reaction profiles were modified from Hrbek et al. ([51]; Additional file 4). Except for *L. vittata*, *P. dominicensis*, and *P. hispaniolana*, the primer combinations L14725 and H15981 were used. 1 µl DNA isolate was used during amplification (increased to 2 µl if DNA concentration was below 20 ng/µl). PCR reactions contained 0.12 µl of 5 U/µl MyTaq mtDNA polymerase (Bioline), 0.5 µl of each 10 µM primer, 5 µl of 5 × MyTaq reaction buffer and HPLC H_2_O up to a final volume of 50 μl. PCR products were sequenced using Applied Biosystems™ BigDye™ Terminator v3.1 Cycle Sequencing Kits (ThermoFisher), purified with ExoSAP (Exonuclease I [59] and Antarctic Phosphatase [60]) according to manuals from New England Biolabs, and run on an Applied Biosystems™ 3500 sequencer.

#### Phylogenetic and haplotype network analyses

Sequences were manually edited and aligned with ClustalW in BioEdit v.7.2 [61, 62] and 1127 bp of cytochrome *b* were used in phylogenetic analyses. Potential mutation saturation was assessed with DAMBE [63]. We conducted Maximum Likelihood analyses using RAxML GUI v.2.0 [64, 65] and assessing clade support via 10,000 rapid bootstrap pseudoreplicates. Separately, we conducted Bayesian analyses in MrBayes v.3.2.7. [66], where we ran four Markov chains for 10,00,000 iterations, sampling every 1000 iterations, with three heated chains and one cold chain and default parameters unlinked across partitions. Convergence was assessed using Tracer v.1.7. All parameter estimates were verified to have been sampled sufficiently (ESS  > 200). We removed the first 25% of our trees as burn-in, such that 3002 trees were retained. Nodes were considered with bootstrap support (BS) and Bayesian posterior probability (PP) greater than 70 and 0.95, respectively [67, 68]. A haplotype network was constructed within PopArt [69] using a median joining network [70]. Genetic distances between taxonomic groups were calculated in MEGA [71].

### Results

**Fig. 1:**
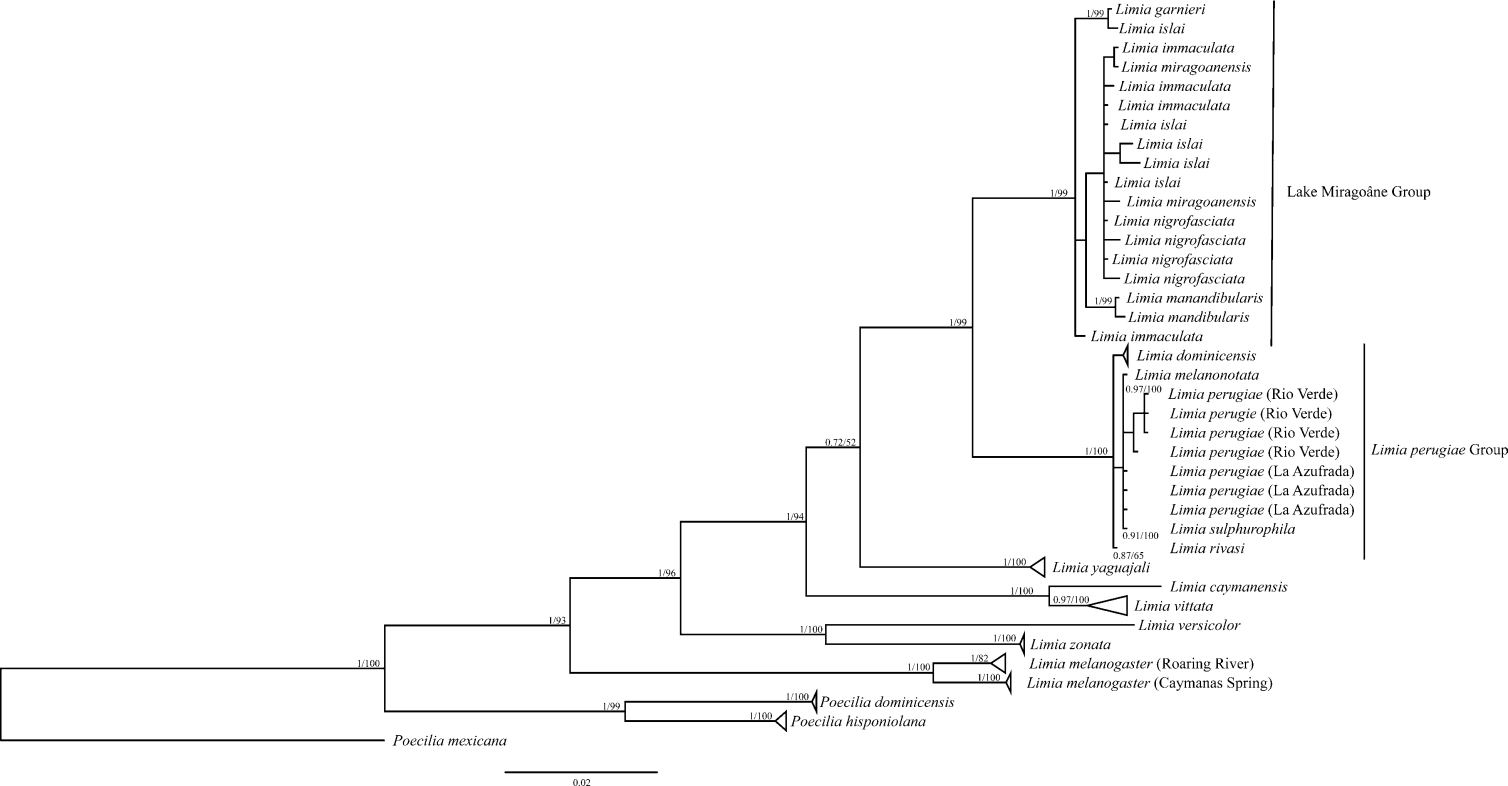
Phylogenetic tree of *Limia* based on the mitochondrial (mt) cytochrome *b* gene. Maximum likelihood bootstrap values are placed after Bayesian inference posterior probabilities at each node. Both phylogenetic analyses revealed identical topologies. Species endemic to Lake Miragoâne formed a separate clade, compatible with an in-situ radiation. Phylogenetic relationships within the Lake Miragoâne clade were not resolved in our analyses

There was no indication of mutation saturation in our data set (Additional file 5). Maximum Likelihood and Bayesian trees revealed nearly identical topologies for interspecific relationships (Fig. 1). In both trees, there is strong support that the species found in Lake Miragoâne in Haiti are monophyletic (BS  = 97; PP  = 1.0). However, within Lake Miragoâne, *L. mandibularis* is the only species resolved as a monophyletic group, while the phenotypically described species *L. islai, L. immacualata, L. miragoanensis*, and *L. nigrofasciata* form a polytomy.

**Fig. 2:**
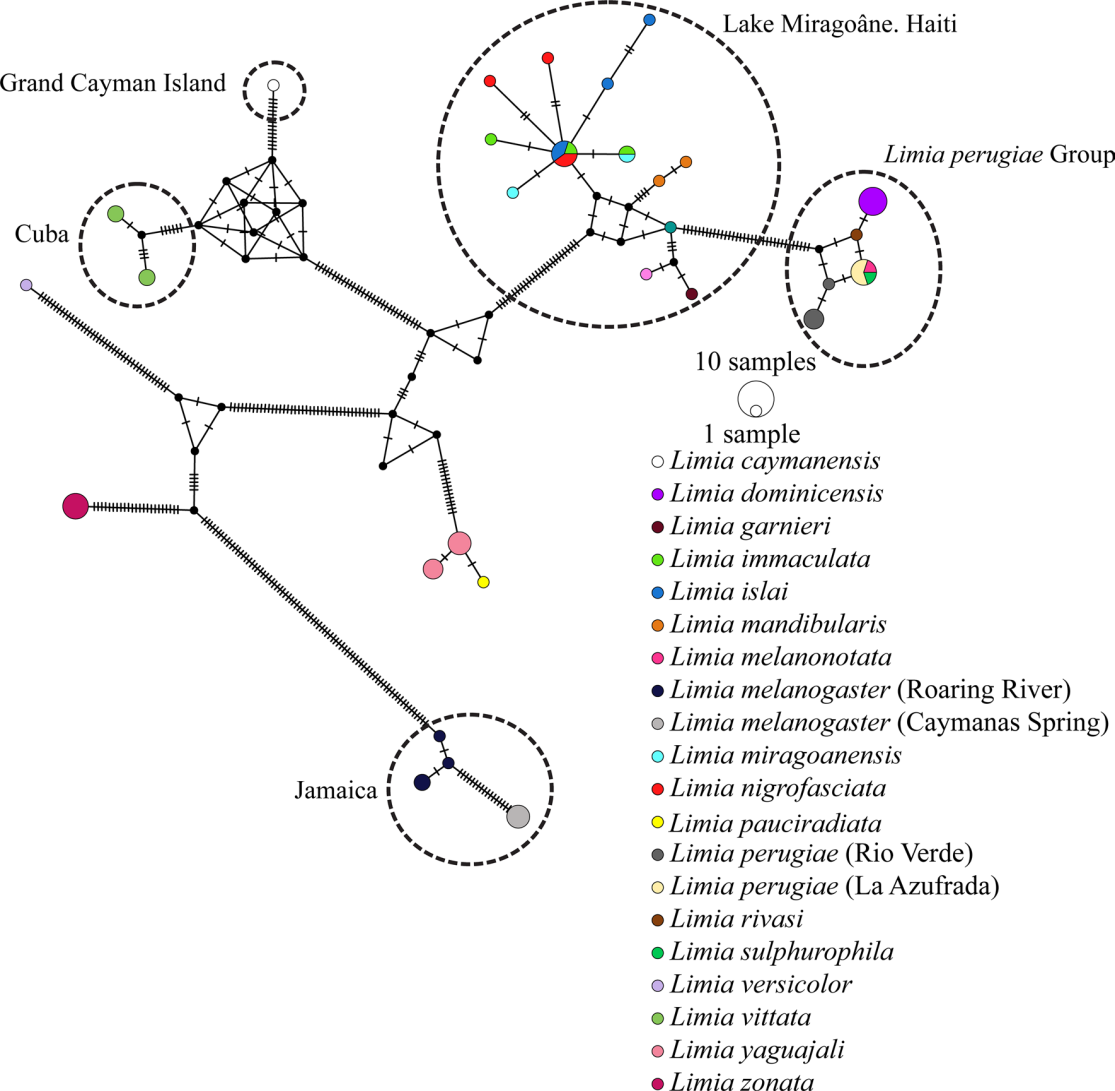
Mitochondrial haplotype network. Both the Lake Miragoâne group and *L. perugiae* group exhibit haplotypes shared among species, indicative of incomplete lineage sorting

For the majority of taxa outside Lake Miragoâne, both species monophylies and respective taxonomic relationships were well supported*. L. yaguajali* is sister to a clade consisting of *L. perugiae*, *L. dominicensis,* and the species in Lake Miragoâne, but this node has only moderate support (BS  = 61; PP  = 72). We found significant genetic divergence among two populations of *L. melanogaster* on Jamaica (BS  = 100; PP  = 1.0; Additional file 6). The haplotype network (Fig. 2) confirms divergent *Limia* evolution among the different Caribbean islands. Within Lake Miragoâne and in the *L. perugiae* group, some mitochondrial haplotypes were shared among morphological species.

### Discussion

**Fig. 3:**
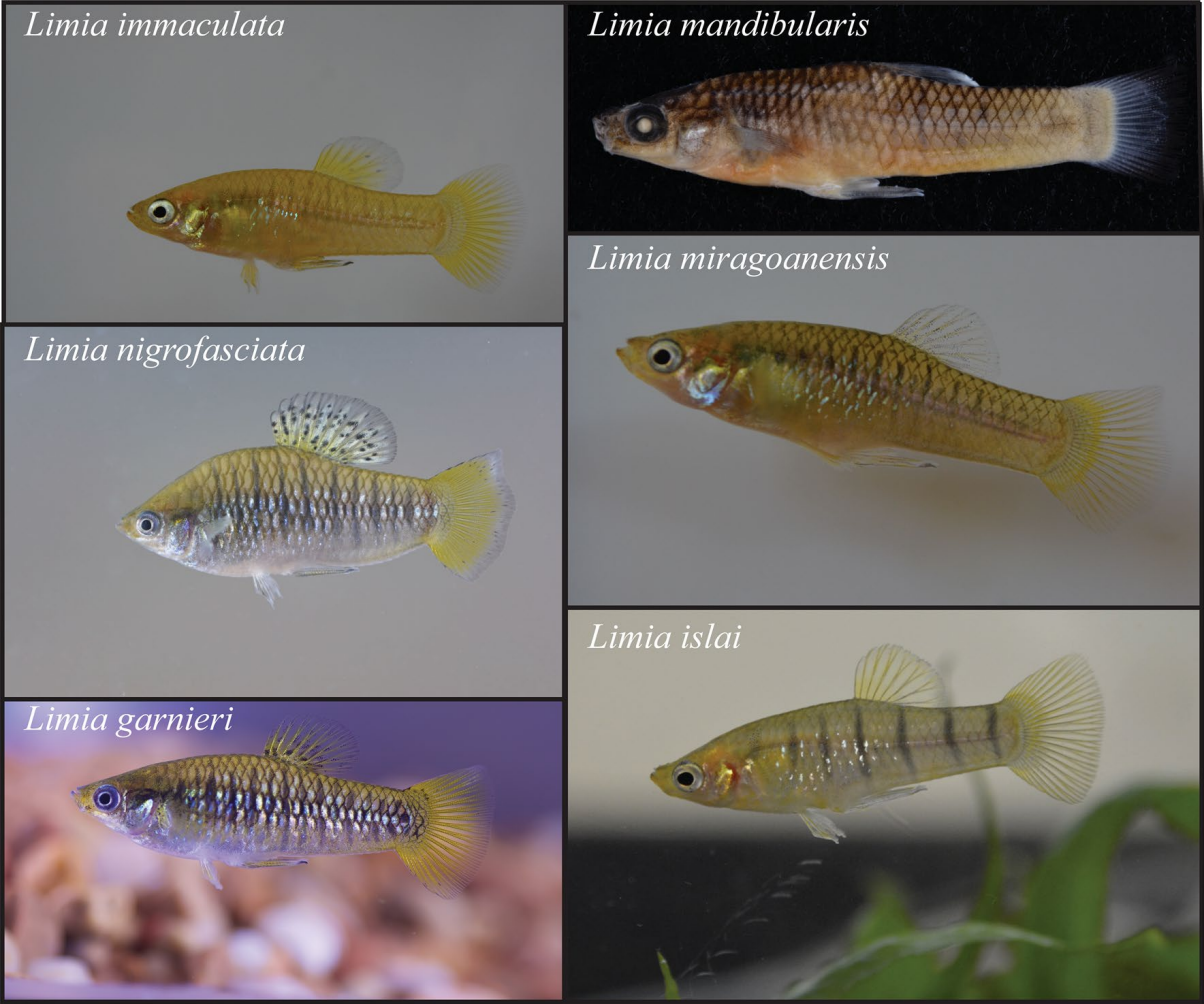
Images of *Limia* found in Lake Miragoâne

The five Limia *species* from Lake Miragoâne are recovered as a well-supported clade in all phylogenetic analyses, indicating an in situ radiation. However, taxonomic relationships within the clade were not resolved. These species have likely diverged too recently for complete lineage sorting and reciprocal monophyly to evolve at a single maternally inherited locus like cytochrome *b*. Alternatively, the observed pattern could be due to species hybridization, introgression, or phenotypic species misassignment [72]. However, the distinct morphological differences of the species found in Lake Miragoâne make us reluctant to attribute the polytomy to phenotypic species misassignment (Fig. 3).

Most other relationships supported in our phylogenetic analyses are consistent with the findings of previous phylogenetic studies [28, 44]. *Limia melanogaster*’s basal placement (Fig. 1) corroborates initially colonization of Jamaica before radiating across other islands in the Greater Antilles [41], lending additional support to the Greater Antilles and Aves Ridge (GAAR) landia hypothesis [44, 73]. The divergence within *L. melanogaster* may be the first evidence of a further cryptic speciation event in *Limia* (Additional file 5). *Limia vittata* from Cuba and *L. caymanensis* from Grand Cayman group within the Hispaniola clade. These two species are likely the sister taxa to *L. yaguajali* which is found in the north of Hispaniola. This coincides with geological evidence that eastern Cuba and north-central Hispaniola were likely connected as a single magmatic arc during the Paleocene-Eocene [74] until the Oligocene [75]. Together, the biogeographic and geological evidence suggests that a *L. vittata* ancestor reached Cuba from the north of Hispaniola and subsequently *L. caymanensis* ancestor reached Grand Cayman from Cuba. Alternatively, they may have reached Cuba via open ocean migration, which has been found in freshwater fishes [5].

The *L. perugiae* group also exhibits a shallow phylogeny with short branches and one haplotype shared across species, again indicating a recent divergence or incomplete lineage sorting. *L. perugiae* is found from hypersaline lagoons to cool freshwater streams and dominates another large lake on Hispaniola, Lake Enriquillo. *Limia perugiae* is also widely distributed throughout Hispaniola with many isolated populations. The combination of *L. perugiae*’s diverse life history strategies and fragmented populations may promote cryptic speciation. However, given our inability to genetically resolve this group, phenotypic plasticity could be an alternative explanation.

It is known that *Limia* species from Lake Miragoâne all inhabit extremely similar niches [76] and they likely possess similar life histories, perhaps with the exception of *L. mandibularis*. This species has well-developed and anteriorly projected lower jaw, deviating from other poeciliids and potentially reflecting specializations in diet [48]. Marked sexual dimorphism, with males such as in *L. nigrofasciata* being extremely ornamented, suggests that sexual selection is also present. Therefore, it is plausible that both natural and sexual selection might—independently or in concert—act as drivers in the potential radiation of *Limia* in Lake Miragoâne.

## Limitations

We present initial evidence for a potential radiation in Lake Miragoâne, yet we recognize the limitations of a single-gene phylogeny. Our preliminary findings are supported by morphometric data that show distinct phenotypic differences between multiple *Limia* populations [1, 43, 45]. The use of multiple unlinked markers, such as microsatellites or SNPs, along with increased population sampling are imperative to understand the radiation event within this clade, as is true for the *L. perugiae* group as well. Such analyses may also resolve the relationship of *L. yaguajali* with *L. vittata* and *L. caymanensis.* We acknowledge that 18 species represent only a subset of the 23 known species of *Limia*, therefore future studies should continue to increase species sampling. Furthermore, our methodology cannot rule out ongoing hybridization between species in Lake Miragoâne, however, the species we keep in the International Stock Center for Livebearing Fishes, appear to breed like regular species.

## Supplementary Information

**Additional file 1: Figure S1. **Biogeographical distribution of *Limia *species on the Greater Antilles. Note the high species number on Hispaniola, suggesting a radiation on that island.

**Additional file 2: Table S1. **Taxon list including collection localities, country of origin, coordinates, date of collection, and accession numbers. ‘NA’ denotes the information is either unavailable or inapplicable.

**Additional file 3: Figure S2. **Sampling sites on Hispaniola. LIM: *Limia immaculata*, LIS: *L. islai*, LMA: *L. mandibularis*, LMI: *L. miragoanensis*, LNI: *L. nigrofasciata*, LPEaz: *L. perugiae* (La Azufrada), LPErv: *L. perugiae* (Rio Verde), LDO: *L. dominicensis*, LVE: *L. versicolor*, LZO: *L. zonata*, LYA: *L. yaguajali*, PDO: *Poecilia dominicensis*, PHI: *P. hispaniolana*.

**Additional file 4: Table S2. **Cytochrome *b *PCR primer pairs and temperature profiles. Primer sequences are listed from 5’ to 3’ ends. For primers not developed during this study, references are provided.

**Additional file 5: Figure S3. **DAMBE saturation plot for our cytochrome *b* data set. There is no indication of saturation, as transisitions (s) exceed transversions (v) and both are linearly correlated to genetic distance.

**Additional file 6: Table S3. **p-distance between genetically analysed species and populations of *Limia* and *Poecilia* based on mitochondrial cytochrome *b*.

## Data Availability

Sequence data are available at Genbank under Accession Numbers MW355516–MW355575.

## Abbreviations

BS: Bootstrap support
CTAB: Cetyl trimethylammonium bromide
GAAR: Greater Antilles and Aves Ridge
*L.*: *Limia*
mt: Mitochondrial
*P.*: *Poecilia*
PCR: Polymerase chain reaction
PP: Bayesian posterior probability
