# Supplementary figures and images for: A phylogeny of the genus Limia (Teleostei: Poeciliidae) suggests a single-lake radiation nested in a Caribbean-wide allopatric speciation scenario
Source: BMC Res Notes. 2021 Nov 25;14:425. doi: 10.1186/s13104-021-05843-x (PMC8613956; doi:10.1186/s13104-021-05843-x)

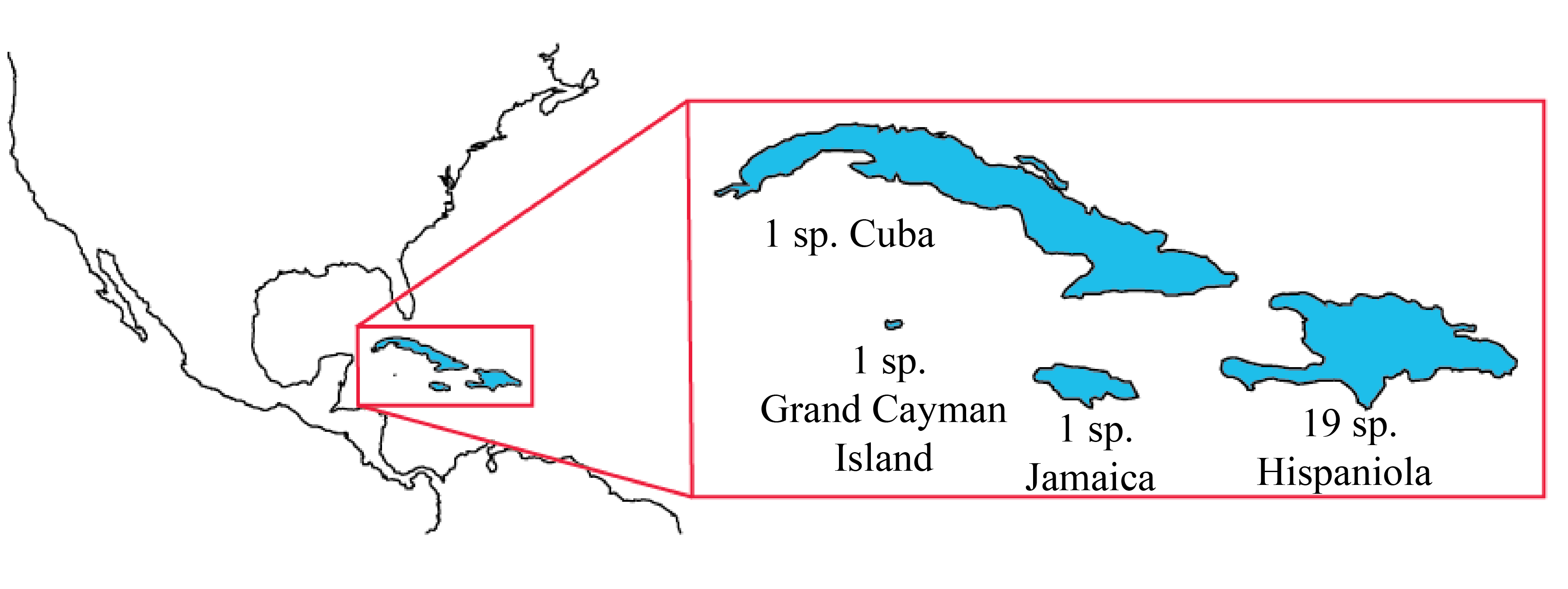

Supplement: Supplementary file 1 — Additional file 1: Figure S1. Biogeographical distribution of Limia species on the Greater Antilles. Note the high species number on Hispaniola, suggesting a radiation on that island. [file 13104_2021_5843_MOESM1_ESM.png]

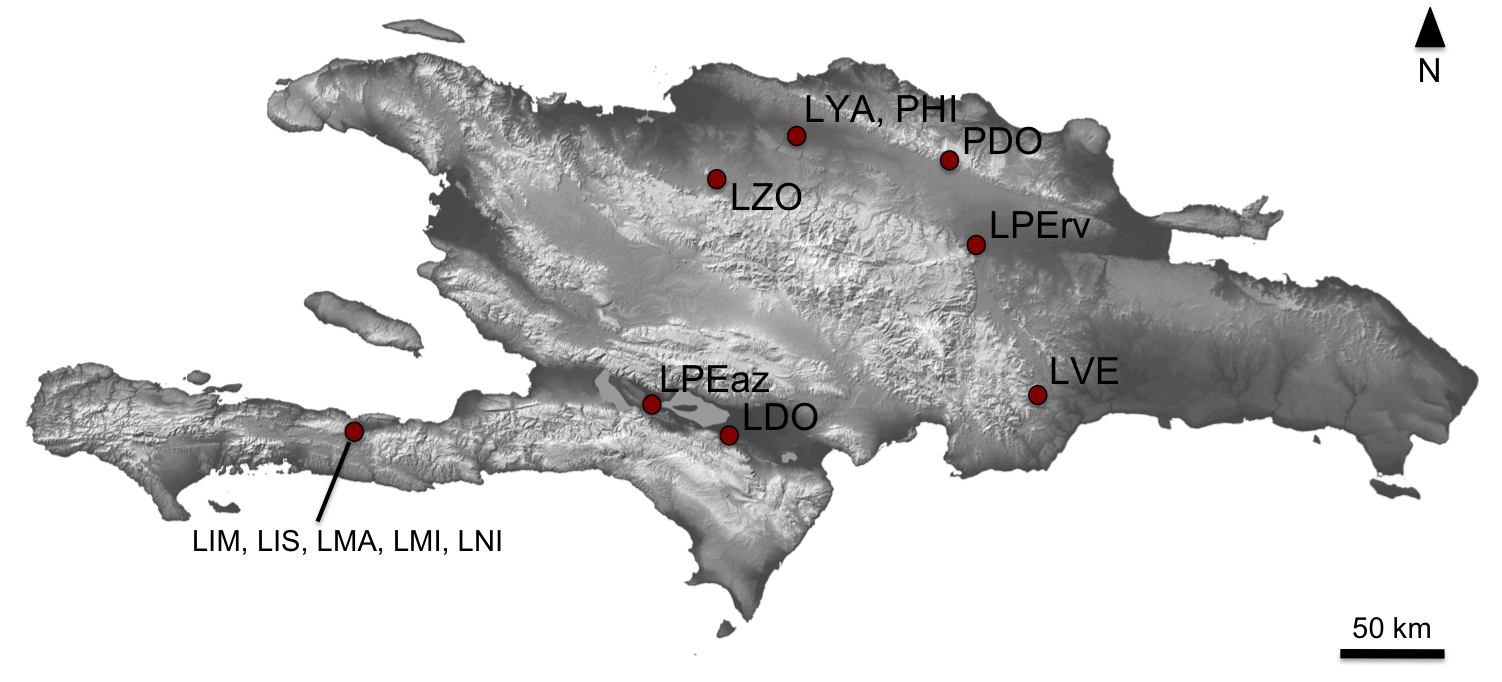

Supplement: Supplementary file 3 — Additional file 3: Figure S2. Sampling sites on Hispaniola. LIM: Limia immaculata, LIS: L. islai, LMA: L. mandibularis, LMI: L. miragoanensis, LNI: L. nigrofasciata, LPEaz: L. perugiae (La Azufrada), LPErv: L. perugiae (Rio Verde), LDO: L. dominicensis, LVE: L. versicolor, LZO: L. zonata, LYA: L. yaguajali, PDO: Poecilia dominicensis, PHI: P. hispaniolana. [file 13104_2021_5843_MOESM3_ESM.tif]

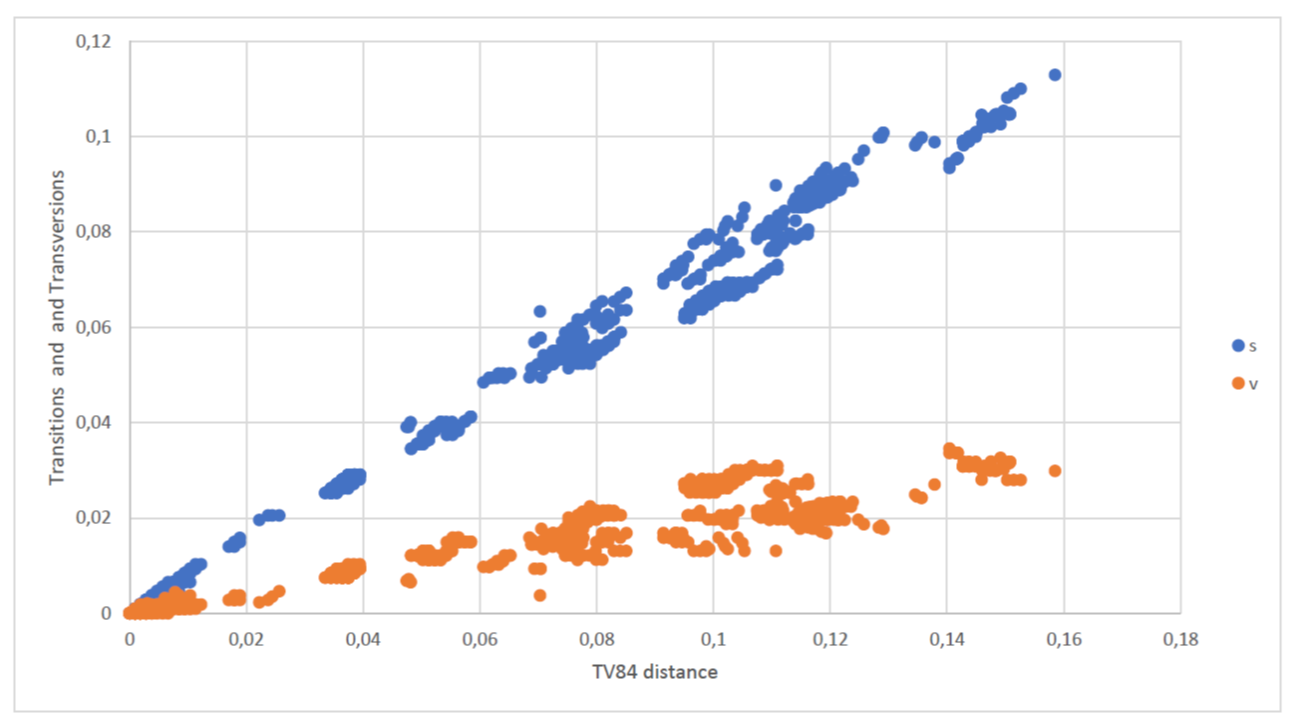

Supplement: Supplementary file 5 — Additional file 5: Figure S3. DAMBE saturation plot for our cytochrome b data set. There is no indication of saturation, as transisitions (s) exceed transversions (v) and both are linearly correlated to genetic distance. [file 13104_2021_5843_MOESM5_ESM.png]
