# Supplementary material for: A phylogeny of the genus Limia (Teleostei: Poeciliidae) suggests a single-lake radiation nested in a Caribbean-wide allopatric speciation scenario
Source: BMC Res Notes. 2021 Nov 25;14:425. doi: 10.1186/s13104-021-05843-x (PMC8613956; doi:10.1186/s13104-021-05843-x)
Supplement: Supplementary file 2 — Additional file 2: Table S1. Taxon list including collection localities, country of origin, coordinates, date of collection, and accession numbers. ‘NA’ denotes the information is either unavailable or inapplicable. [file 13104_2021_5843_MOESM2_ESM.pdf]

Table 1. Taxon list including collection localities, country of origin, coordinates, date of collection, and accession numbers. 'NA' denotes the information is either unavailable or inapplicable.

Species	No. of Individuals	Locality	Latitude	Longitude	Date of Collection	Accession Number	Reference
*Limia caymanensis**	1	NA. Grand Cayman Island	NA	NA	Sep-1991	KJ696810.1	(50)
Limia dominicensis	6	Puerto Escondido. DR	18.319	−71.570	Mar-2014	MW355516 - 21	NA
*Limia garnieri**	1	NA. DR	NA	NA	NA	KJ696811.1	(50)
Limia immaculata	4	Lake Miragoâne. Haiti	18.426	−73.049	Jun-2019	MW355522 - 25	NA
Limia islai	5	Lake Miragoâne. Haiti	18.426	−73.049	Jun-2019	MW355526 - 30	NA
Limia mandibularis	2	Lake Miragoâne. Haiti	18.426	−73.049	Jun-2019	MW355531 - 32	NA
Limia melanogaster	4	Caymanas Spring. Jamaica	18.042	−76.910	Jun-2017	MW355533 - 36	NA
Limia melanogaster	4	Roaring River. Jamaica	18.283	−78.056	Jun-2017	MW355537 - 40	NA
*Limia melanononata**	1	Calion Papito. DR	NA	NA	Apr-1949	KJ696813.1	(50)
Limia miragoanensis	2	Lake Miragoâne. Haiti	18.426	−73.049	Jun-2019	MW355541 - 42	NA
Limia nigrofasciata	4	Lake Miragoâne. Haiti	18.426	−73.049	Jun-2019	MW355543 - 46	NA
*Limia pauciradiata**	1	Lake Miragoâne. Haiti	NA	NA	Jan-1986	KJ696815.1	(50)
Limia perugiae	4	Rio Verde. DR	19.312	−70.524	Dec-2018	MW355547 - 50	NA
Limia perugiae	3	La Azufrada. DR	18.561	−71.700	Jun-2003	MW355551 - 53	NA
*Limia rivasi**	1	NA. DR	NA	NA	NA	KJ696817.1	(50)
*Limia sulphurophila**	1	NA. DR	NA	NA	NA	KJ696818.1	(50)
*Limia versicolor**	1	Rio Haina. DR	NA	NA	Jun-1977	KJ696819.1	(50)
Limia vittata	4	Guanimar. Cuba	22.694	−82.651	Aug-2018	MW355554 - 57	NA
Limia yaguajali	7	Rio Cana. DR	19.496	−71.281	May-2019	MW355558 - 64	NA
Limia zonata	5	Rio Yaguajal	19.472	−70.342	Dec-2018	MW355565 - 69	NA
Poecilia dominicesis	3	Rio Yasica Abajo. DR	19.633	−70.594	2018	MW355570 - 71	NA
Poecilia hispaniolana	4	Rio Cana. DR	19.496	−71.280	May-2019	MW355572 - 75	NA
*Poecilia mexicana**	1	Cueva Luna Azufre. Mexico	17.442	−92.773	Jan-2006	EU269065.1	(56)

* Sequences acquired from GenBank
