# Supplementary material for: A phylogeny of the genus Limia (Teleostei: Poeciliidae) suggests a single-lake radiation nested in a Caribbean-wide allopatric speciation scenario
Source: BMC Res Notes. 2021 Nov 25;14:425. doi: 10.1186/s13104-021-05843-x (PMC8613956; doi:10.1186/s13104-021-05843-x)
Supplement: Supplementary file 4 — Additional file 4: Table S2. Cytochrome b PCR primer pairs and temperature profiles. Primer sequences are listed from 5’ to 3’ ends. For primers not developed during this study, references are provided. [file 13104_2021_5843_MOESM4_ESM.pdf]

Table 2. List of cytochrome *b* PCR primer pairs and temperature profiles. Primer sequences are listed from 5’ to 3’ ends. References are provided where applicable.

Primer pairs	Sequence	Temperature profiles	Reference
Forward: L14725<br>Reverse: H15981	5'-GAYTTGAARAACCAYCGTTG-3'<br>5’-GAATYCTAGCTTTGGGAGYTAG-3'	1 cycle of 1 min at 95 °C<br>40 cycles 25 s at 95 °C<br>20 s at 46 °C 25 s at 72 °C<br>1 cycle 10 min at 72 °C	(48)
Forward: Limia L14740<br>Reverse: Limia-Hint	5'-CCGTTGTWAYTCAACTCACAAG-3'<br>5'-GGTGAAGTTTTCTGGGTCTCC-3'	1 cycle of 1 min at 95 °C<br>40 cycles 25 s at 95 °C<br>20 s at 46 °C 25 s at 72 °C<br>1 cycle 10 min at 72 °C	(48)
Forward: Limia Lint<br>Reverse: Limia H15981	5'-GCCTYGTMCAATGAATCTGAGG-3'<br>5'-GAATYCTAGCTTTGGGAGYTAG-3'	1 cycle of 1 min at 95 °C<br>40 cycles 25 s at 95 °C<br>20 s at 46 °C 25 s at 72 °C<br>1 cycle 10 min at 72 °C	-
Forward: Limia Lint<br>Reverse: Limia H16249	5'-GCCTYGTMCAATGAATCTGAGG-3'<br>5'-GCCGACTTTCGATTTACAAGAC-3'	1 cycle of 1 min at 95 °C<br>40 cycles 25 s at 95 °C<br>20 s at 46 °C 25 s at 72 °C<br>1 cycle 10 min at 72 °C	-
Forward: Limia L14763<br>Reverse: Limia Hint	5'-GACCTACGAAAATCYCACCCCC-3'<br>5'-GGTGAAGTTTTCTGGGTCTCC-3'	1 cycle of 1 min at 95 °C<br>40 cycles 25 s at 95 °C<br>20 s at 50 °C 25 s at 72 °C<br>1 cycle 10 min at 72 °C	-
