# Supplementary material for: A phylogeny of the genus Limia (Teleostei: Poeciliidae) suggests a single-lake radiation nested in a Caribbean-wide allopatric speciation scenario
Source: BMC Res Notes. 2021 Nov 25;14:425. doi: 10.1186/s13104-021-05843-x (PMC8613956; doi:10.1186/s13104-021-05843-x)
Supplement: Supplementary file 6 — Additional file 6: Table S3. p-distance between genetically analysed species and populations of Limia and Poecilia based on mitochondrial cytochrome b. [file 13104_2021_5843_MOESM6_ESM.pdf]

Supplemental Table 1. Estimates of evolutionary divergence over sequence pairs between groups p-distance between genetically analysed species and populations of Limia and Poecilia based on mitochondrial cytochrome *b*.

Species	*Limia caymanensis*	*Limia dominicensis*	*Limia garnieri*	*Limia immaculata*	*Limia islai*	*Limia mandibularis*	*Limia melanononata*	*Limia melanogaster* (C)	*Limia melanogaster* (R)	*Limia miragoanensis*	*Limia nigrofasciata*	*Limia perugiae* (V)	*Limia perugiae* (A)	*Limia rivasi*	*Limia sulphurophila*	*Limia versicolor*	*Limia vittata*	*Limia yaguajali*	*Limia zonata*	*Poecilia dominicensis*	*Poecilia hispaniolana*	*Limia pauciradiata*	*Poecilia mexicana*
Limia caymanensis	NA	NA	NA	NA	NA	NA	NA	NA	NA	NA	NA	NA	NA	NA	NA	NA	NA	NA	NA	NA	NA	NA	NA
Limia dominicensis	0.071	NA	NA	NA	NA	NA	NA	NA	NA	NA	NA	NA	NA	NA	NA	NA	NA	NA	NA	NA	NA	NA	NA
Limia garnieri	0.074	0.036	NA	NA	NA	NA	NA	NA	NA	NA	NA	NA	NA	NA	NA	NA	NA	NA	NA	NA	NA	NA	NA
Limia immaculata	0.071	0.036	0.006	NA	NA	NA	NA	NA	NA	NA	NA	NA	NA	NA	NA	NA	NA	NA	NA	NA	NA	NA	NA
Limia islai	0.072	0.038	0.006	0.003	NA	NA	NA	NA	NA	NA	NA	NA	NA	NA	NA	NA	NA	NA	NA	NA	NA	NA	NA
Limia mandibularis	0.074	0.038	0.009	0.006	0.007	NA	NA	NA	NA	NA	NA	NA	NA	NA	NA	NA	NA	NA	NA	NA	NA	NA	NA
Limia melanononata	0.072	0.002	0.036	0.036	0.038	0.038	NA	NA	NA	NA	NA	NA	NA	NA	NA	NA	NA	NA	NA	NA	NA	NA	NA
Limia melanogaster (C)	0.102	0.101	0.094	0.092	0.092	0.095	0.101	NA	NA	NA	NA	NA	NA	NA	NA	NA	NA	NA	NA	NA	NA	NA	NA
Limia melanogaster (R)	0.107	0.105	0.096	0.094	0.094	0.097	0.105	0.017	NA	NA	NA	NA	NA	NA	NA	NA	NA	NA	NA	NA	NA	NA	NA
Limia miragoanensis	0.071	0.037	0.008	0.002	0.003	0.007	0.037	0.092	0.094	NA	NA	NA	NA	NA	NA	NA	NA	NA	NA	NA	NA	NA	NA
Limia nigrofasciata	0.071	0.037	0.008	0.002	0.003	0.007	0.037	0.092	0.094	0.002	NA	NA	NA	NA	NA	NA	NA	NA	NA	NA	NA	NA	NA
Limia perugiae (V)	0.070	0.004	0.036	0.036	0.038	0.038	0.002	0.100	0.104	0.037	0.037	NA	NA	NA	NA	NA	NA	NA	NA	NA	NA	NA	NA
Limia perugiae (A)	0.072	0.002	0.036	0.036	0.038	0.038	0.000	0.101	0.105	0.037	0.037	0.002	NA	NA	NA	NA	NA	NA	NA	NA	NA	NA	NA
Limia rivasi	0.071	0.001	0.035	0.035	0.037	0.037	0.001	0.100	0.104	0.036	0.037	0.003	0.001	NA	NA	NA	NA	NA	NA	NA	NA	NA	NA
Limia sulphurophila	0.072	0.002	0.036	0.036	0.038	0.038	0.000	0.101	0.105	0.037	0.037	0.002	0.000	0.001	NA	NA	NA	NA	NA	NA	NA	NA	NA
Limia versicolor	0.102	0.094	0.090	0.088	0.089	0.090	0.094	0.097	0.102	0.088	0.088	0.092	0.094	0.093	0.094	NA	NA	NA	NA	NA	NA	NA	NA
Limia vittata	0.023	0.074	0.071	0.068	0.069	0.071	0.075	0.101	0.106	0.068	0.068	0.077	0.075	0.074	0.075	0.096	NA	NA	NA	NA	NA	NA	NA
Limia yaguajali	0.066	0.055	0.050	0.048	0.049	0.052	0.055	0.096	0.102	0.049	0.049	0.057	0.055	0.054	0.055	0.092	0.060	NA	NA	NA	NA	NA	NA
Limia zonata	0.098	0.080	0.078	0.078	0.078	0.081	0.080	0.099	0.102	0.079	0.078	0.080	0.080	0.079	0.080	0.067	0.094	0.080	NA	NA	NA	NA	NA
Poecilia dominicensis	0.115	0.111	0.109	0.111	0.112	0.113	0.111	0.109	0.109	0.110	0.112	0.109	0.111	0.110	0.111	0.117	0.111	0.112	0.108	NA	NA	NA	NA
Poecilia hispaniolana	0.109	0.107	0.107	0.108	0.109	0.111	0.107	0.106	0.110	0.108	0.110	0.107	0.107	0.106	0.107	0.119	0.106	0.106	0.109	0.047	NA	NA	NA
Limia pauciradiata	0.066	0.056	0.050	0.049	0.050	0.053	0.056	0.095	0.102	0.049	0.049	0.058	0.056	0.055	0.056	0.091	0.061	0.001	0.082	0.112	0.107	NA	NA
Poecilia mexicana	0.132	0.130	0.135	0.131	0.132	0.136	0.131	0.133	0.138	0.131	0.131	0.131	0.131	0.130	0.131	0.143	0.136	0.139	0.136	0.128	0.126	0.138	NA
